# An evaluation of bird weight and diet nutrient density during early lay on ISA Brown performance, egg quality, bone characteristics, and liver health at 50 weeks of age

**DOI:** 10.1016/j.psj.2022.101765

**Published:** 2022-01-30

**Authors:** Wendy I. Muir, Yeasmin Akter, Kenneth Bruerton, Peter J. Groves

**Affiliations:** ⁎ School of Life and Environmental Science, Poultry Research Foundation, Faculty of Science, The University of Sydney, Camden, NSW 2570, Australia; † PO Box 1362, Elanora, QLD 4221, Australia; ‡ Sydney School of Veterinary Science, Poultry Research Foundation, Faculty of Science, The University of Sydney, Camden, NSW 2570, Australia.

**Keywords:** hen weight, diet nutrient density, eggshell quality, liver health, feed conversion ratio

## Abstract

This study compared the impact of a higher nutrient density (**HND**) or lower nutrient density (**LND**) diet fed during early lay to either heavier weight (**HW**) or lighter weight (**LW**) ISA Brown hens. At 18 wk of age (**WOA**) pullets (n = 240) were evenly assigned to either HW (n = 120) or LW (n = 120). Sixty birds from each weight group were then randomized to either the HND or LND diet treatments which were fed from 18 to 24 WOA inclusive. At 25 WOA the LND diet replaced the HND diet. All hens remained on LND diet to 50 WOA. Hen performance was measured from 18 to 50 WOA. Femur and liver health were evaluated at 50 WOA. Egg quality was assessed from 46 to 50 WOA. The 18 WOA HW hens had higher BW, cumulative egg production, cumulative feed intake (**CFI**), and cumulative egg mass (**CEM**) to both 24 and 50 WOA (*P* < 0.01). At 24 WOA the HND diet also generated higher BW (*P <* 0.001), CEM (*P* < 0.001) and lower cumulative feed conversion ratio (**CFCR**) (*P* < 0.01), the latter being sustained to 50 WOA (*P* < 0.01). At 50 WOA CFCR of LW birds was lower than HW birds (*P <* 0.01). Egg weight (**EW**), yolk diameter, and percent yolk weight were higher (*P* < 0.05) in the HW birds with the highest albumen to yolk ratio in LW birds (*P* < 0.05). Egg shape index was higher in LND diet fed birds (*P* < 0.01) while LW hens had higher shell phosphorus (*P* < 0.05). Body weight and diet nutrient density interacted on femoral diameter and cortical thickness being higher (*P <* 0.01) in LW birds fed HND than LW birds fed LND diets. Fatty liver hemorrhagic scores (*P <* 0.05) and liver lipid peroxidase (*P* < 0.001) at 50 WOA were higher in HW and LND diet treatments. Concurrently HW birds had the highest CFI and EW while CFCR and liver health were superior in LW and the HND diet treatment.

## INTRODUCTION

The development of modern brown eggshell laying strains of hens capable of high productivity has been a primary goal of commercial poultry breeders. However, the characteristics of larger compared to smaller sized layer pullets creates contention around the most appropriately sized pullet to bring to point of lay (**POL**). Lighter pullets have a lower maintenance cost in part due to their lower feed intake (**FI**) but are slower to reach sexual maturity (Summers et al., 1991) and, as egg weight (**EW)** is aligned with body weight **(BW**) at sexual maturity (Robinson and Sheridan, 1982; Summers and Leeson, 1983), their average egg size is also smaller. On the contrary, larger-sized pullets tend to reach sexual maturity earlier and ultimately lay larger sized eggs. Further, larger hens are less likely to experience cloacal hemorrhage, prolapse, and oviduct infection leading to peritonitis (Cransberg and Parkinson, 2006). They are also generally more resilient throughout transport and transition to the layer facility than smaller sized pullets (P.J Groves, The University of Sydney, Camden, NSW, personal communication). These factors have driven the rearing industry to raise larger POL pullets, where the average BW of Australian layer hens are often between 100 and 300 g above the recommended breed standard weight (**BSW**) for age (Parkinson et al., 2015). But there may also be disadvantages to heavier POL pullets and hens including a poorer continuity of lay and cumulative egg production (**EP**) and, reduced eggshell quality as they age (Parkinson et al., 2015). Heavier birds also demonstrate poorer feed efficiency, where high feed efficiency layer hens tend to be the lighter BW (**LW**) birds (Akter et al., 2019). Hence pullet size at POL presents a double-edged sword and tailored management of POL pullets of both heavier and lighter weights may offer opportunities to improve bird production and egg quality. This may be particularly important as EP moves toward the longer laying cycle.

The global layer industry, including Australia's egg industry, are pursuing the extension of layer hen productive life to 100 wk of age (**WOA**), which could deliver benefits for the environment and overall industry sustainability (Dunn, 2013). For this to be successful mechanisms for supporting longer term hen productivity, hen health, hen welfare, and eggshell quality are critical (Bain et al., 2016). Strategies designed to sustain these characteristics require initial assessment during the early to mid-laying phases allowing the identification of processes that may prepare birds for success in extended laying cycles.

Good bird health and welfare, including bone and liver health, are critical for the laying hen. For example, the ongoing demand for Ca for eggshell production in hens in an extended laying cycle may interplay with bone integrity (Alfonso-Carillo et al., 2021) and can increase susceptibility to osteoporosis (Whitehead and Fleming, 2000). The high rate of lipid metabolism required during egg formation may result in fatty liver hemorrhagic syndrome (**FLHS**) (Yang et al., 2017) and sudden bird mortality. Fatty liver hemorrhagic syndrome has been more commonly observed in birds of higher feed and energy intake (Shini et al., 2020b), in cage layer systems (Shini et al., 2019). Hence bone and liver health are important aspects of bird wellbeing that should be assessed when considering extending hen production.

On initial consideration the more efficient smaller sized hens look well suited to a longer laying cycle. However, as the lighter birds tend to have lower FI then their larger counterparts (Harms et al., 1982) there is uncertainty as to whether they can consume sufficient diet to meet their nutritional needs, especially when the diet has been formulated on the BSW hen average daily feed intake (**ADFI**; Leeson et al., 2001). To meet their nutritional requirements birds may alter their ADFI in response to the diet nutrient density (Harms et al., 2000; Zhang and Kim, 2013). Therefore, the formulation of a higher nutrient density (**HND**) diet could be used to counterbalance the different levels of feed and nutrient intake in different sized birds. A HND diet may also encourage appropriate nutritional partitioning and improve feed efficiency, greater continuity in egg production, and eggshell quality through to mid-lay. Several studies have investigated the relationship between diet nutrient density, ADFI, and bird performance, predominantly in white laying hens (Latshaw et al., 1990; Leeson et al., 2001; Ribeiro et al., 2014; dePersio et al., 2015) with few reports on these relationships in current day brown egg layer hens (Perez-Bonilla et al., 2012), and one study that assessed both brown and white shell layers (Harms et al., 2000).

As close management of the early lay diet could provide a nutritional prime for the laying period (dePerisio et al., 2015), its use is worthy of exploration with current day brown egg layers. Therefore, this study was designed to compare hen productivity, cumulative feed efficiency and EP, eggshell, and bone quality together with liver health, through to 50 WOA in ISA Brown pullets of different mean BW at POL that had been fed either a HND or lower nutrient density (**LND**) diet from 18 to 24 WOA.

## MATERIALS AND METHODS

### Ethical Approval

This work was conducted at the Poultry Research Unit, The Sydney of University, Camden campus. All experimental procedures were approved by the University of Sydney Animal Ethics Committee (Protocol 2019/1623) and were in accordance with the Australian code for the care and use of animals for scientific purposes (8th Edition, National Health and Medical Research Council, 2013).

### Experimental Design

This study was a 2 × 2 factorial arrangement comparing 18 WOA bird BW and diet nutrient density. Specifically, two 18 WOA BW groups, that is Heavier weight (**HW**) with mean weight 1.65 kg and LW with mean body weight 1.49 kg, were fed either a diet of LND or HND to 24 WOA. Relative to the breed standard weight (**BSW**) 120 heavier weight and 120 lighter weight ISA Brown pullets were purchased at 16 WOA from a commercial grower and housed individually in 25 × 50 × 50 cm unfurnished cages within an environmentally controlled high-rise layer shed at the Poultry Research Unit, The University of Sydney, Camden Campus, Australia. Between 16 and 18 WOA all birds were fed a LND diet ad libitum while acclimatizing to the experimental facility.

At 18 WOA all 240 hens were weighed, and 120 pullets allocated to either the HW or LW body weight treatment groups. Within each body weight treatment group 60 pullets were then randomly allocated to the experimental dietary treatments of either a HND diet, formulated for 90 g ADFI (2,901 kcal/kg, 0.83% Standardized ileal digestible [**SID**] Lysine) or LND diet, formulated for 110 g ADFI (2,726 kcal/kg, 0.737% SID of Lysine) (Table 1). The LND diet formulation was based on the observed ADFI for peak egg production by ISA Brown hens fed a similar LND diet in the same research facility (Akter et al., 2019), in conjunction with the ISA Brown Management Nutrition Guide (2019). The HND diet was then formulated for 20 g lower ADFI (Table 1). Hence there were four treatment groups of 60 birds each: HW birds fed HND diet, HW bird fed LND diet, LW birds fed HND diet and LW birds fed LND diet.

**Table 1:** Ingredients and nutrient composition of early and mid-lay experimental diets.

		Early-lay diet		Mid-lay diet	
		HND^1^	LND^2^		LND^3^
Ingredients	(% protein)	(90 g/d^4^)	(110 g/d^4^)	(% protein)	(>110 g/d^4^)
Sorghum	11.0	300.00	300.00	9.9	355.00
Wheat	12.5	353.14	402.64	15.8	363.79
Soybean	47.5	192.00	107.00	46.0	50.00
Lime grit	38.0	65.00	75.00	38.0	78.00
Soybean oil		32.00	7.00		6.00
Limestone		25.00	25.00		25.00
Dicalcium phosphate		12.00	5.00		3.00
Canola Sol	38.0	10.00	69.00	38.0	110.0
Sodium bicarbonate		2.80	2.70		2.90
DL-methionine		2.40	1.55		1.20
Salt		1.60	1.40		1.20
Lysine - HCl		1.50	1.70		2.05
Layer pre-mix^5^		1.00	1.00		1.00
L-Threonine		0.50	0.30		0.20
Choline chloride	60.0	0.50	0.50	60.0	0.50
L-Valine		0.40	0.05		0.00
AXTRA XB 201		0.10	0.10		0.10
AXTRAPHY TPT 100		0.06	0.06		0.06
Total		1,000	1,000		1,000
Calculated value					
ME-enzyme (kcal/kg)		2,901.32	2,726.31		2724.20
NE Layer (kcal/kg)		2,255.28	2,078.46		2077.17
Crude protein (%)		17.625	16.377		16.023
Lysine (%)		0.893	0.804		0.763
Methionine (%)		0.492	0.406		0.377
Methionine & cystine (%)		0.789	0.710		0.690
Threonine (%)		0.654	0.587		0.558
Isoleucine (%)		0.700	0.625		0.591
Leucine (%)		1.459	1.348		1.304
Tryptophan (%)		0.218	0.202		0.193
Arginine (%)		1.022	0.886		0.813
Stand. ileal digest Lys. (%)		0.83	0.737		0.695
Crude fat (%)		4.916	2.54		2.532
Linoleic acid (%)		2.613	1.315		1.297
Total xanthophylls (mg/kg)		6.00	6.00		6.00
Red xanthophylls (mg/kg)		3.10	3.10		3.10
Yellow xanthophyl (mg/kg)		2.90	2.90		2.90
Ash (%)		13.051	13.31		13.369
Calcium (%)		3.981	4.212		4.289
Available phosphorus (%)		0.446	0.347		0.314
Total phosphorus (%)		0.556	0.445		0.419
Sodium (%)		0.178	0.17		0.169
Chloride (%)		0.178	0.173		0.170
Choline mg/kg)		1,274.28	1,163.5		1,028.714
ME enzyme (MJ/kg)		12.412	11.41		11.401
NE layer (MJ/kg)		9.438	8.698		8.693
Analyzed value					
GE (MJ/kg)		15.60	14.86		14.3
Crude protein (%)		17.9	15.7		16.2
Crude fat (%)		3.1	2.1		2.7
Ca (%)		5.43	6.20		5.05
P (%)		0.57	0.40		0.46

1 Early-lay HND: Early-lay higher nutrient density diet.

2 Early-lay LND: Early lay lower nutrient density diet.

3 Mid-lay LND: Mid-lay lower nutrient density diet.

4 Average daily feed intake used for formulation.

5 Layer premix composition/kg: Vitamin D3: 3.5 MIU; Vitamin A: 10 MIU; Vitamin E: 30 g; Vitamin K3: 3 g; Vitamin B1: 2.5 g; Vitamin B2: 5.5 g; Vitamin B3: 30 g; Vitamin B5: 9 g; Vitamin B6: 4 g; Vitamin B12: 0.2 g; Biotin H: 0.15 g; Copper: 8 g; Iodine: 1.5 g; Selenium: 0.25 g; Iron: 50 g; Zinc: 60 g; Manganese: 60 g; Carophyll Red 10%: 3.1 g; Carophyll Yellow 10%: 2.9 g; Ethoxyquin: 75 g.

The hens were fed their allocated experimental diet (HND or LND) from 18 to 24 WOA inclusive. At 25 WOA hens on the HND diet were consuming an average 100 g/d. As this was an additional 10 g feed/d compared to the diet formulation all birds on the HND diet were changed to the LND diet. This was to meet the experimental aim of assessing the HND diet as a nutritional primer rather than as an ongoing feeding option. From that point all birds were fed a LND diet. When birds were 40 WOA the LND diet was changed from the Early-lay to Mid-lay diet formulated to >110 g ADFI (2,724 kcal/kg, 0.695% SID Lysine; Table 1). Birds remained on that diet until they were 50 WOA. Each bird had access to an individual feeder, waterer, and pecking string with the diet being provided ad libitum as a mash.

### Experimental Diets

The formulations of the experimental diets are shown in Table 1, together with the analyzed gross energy (**GE**), crude protein (**CP**), crude fat, calcium (**Ca**), and phosphorus (**P**) of the mixed diets after grinding. The gross energy was assessed using a Parr 1280 adibatic bomb calorimeter (Parr Instrument Co, Moline, IL) at The University of Sydney, Poultry Research Laboratory, Camden, Australia. The CP content was determined by Dumas method using a Leco FP-528 (Leco Corporation, St. Joseph, MI.) (Sweeney, 1989) and the crude fat by modified Randall system, where the petroleum ether was evaporated at 105°C instead of 102°C using Velp Scientifica, SER 148 solvent extraction unit (Usmate Velate, Monza and Brianza, Lombarda, Italy) (AOAC Official Method of Analysis, Method 2003.05 Method 2006, 2022) at Birling Avian Laboratories, Bringelly, Australia. The Ca and P content of the diets was determined at the University of New South Wales by inductively coupled plasma optical emission spectrometry (**ICP**) using a PerkinElmer OPTIMA 7300 (PerkinElmer Inc., Waltham, MA) following digestion with nitric acid and hydrogen peroxide as described by Hopcroft et al. (2020).

### Body Weight and Production Performance to 50 Wk of Age

Hens were weighed at 18, 24, and 50 WOA. Across that experimental period FI, EP, and EW were recorded. Feed intake was calculated weekly for individual hens as feed offered minus feed remaining and cumulative FI (**CFI**) from 18 to 24 and 18 to 50 WOA inclusive, was calculated. Egg production was recorded daily for each hen and was computed weekly as: (n / 7) × 100, where n = number of eggs laid/hen in 7 d. The total number of eggs produced by each hen was recorded between 18 and 50 WOA to determined cumulative EP (**CEP**). Eggs were collected daily, weighed using an electronic scale with a digital output, accurate to 1 g, and the weekly average EW/hen was determined. Egg mass (**EM**) /hen/wk was then calculated as: EP × EW. The CFI as kg per kg of cumulative EM (**CEM**) was then used to calculate cumulative feed conversion ratio (**CFCR**) for each bird and each treatment group across the 18 to 50 WOA period.

### Egg Quality

For each treatment group 12 hens were chosen at random for assessment of egg quality from 46 to 50 WOA. The fresh egg was collected from each of these birds on the same day each week for internal egg quality and eggshell assessment. Prior to egg break out EW was measured using an electronic weighing scale while egg height (length) and width (diameter at the equator) were measured using a 200-mm digital Vernier caliper (Kincrome, Australia). Egg shape index (**ESI**) was then determined as egg width/egg height multiplied by 100 (Anderson et al., 2004). For internal egg quality assessment eggs were broken out onto a flat, level glass surface on a metal stand positioned above a reflective mirror. The height of the thick albumen was measured using an albumen height gauge (Technical Services and Supplies, York, United Kingdom). The Haugh unit was calculated using the formula 100 × log_10_ (*h* - 1.7 × *w*^0.37^ + 7.6), where *h* = albumen height (mm), *w* = EW (g) (Monira et al., 2003) and, yolk width (diameter) was measured using a 200-mm digital Vernier caliper (Kincrome, Australia). Yolk color score was determined using a DSM Yolk Color Fan, (DSM, Switzerland, 2005), with the range from 1 (pale yellow) through to 15 (deep orange) color scale. Using a plastic scrapper, the albumen and yolk were carefully separated and weighed and then the ratio of albumen to yolk was calculated. For eggshell characteristics the eggshell (without membranes) was gently washed, air dried, and weighed with a digital scale. Egg shell thickness was calculated as the average thickness measured at the top, equator and base of the egg using a 200-mm digital Vernier caliper. On the subsequent day fresh eggs were collected from the same hens to measure eggshell breaking strength. Egg shell breaking strength (N) was determined at the broad end of the egg using a 3-point bending test to identify the peak force to fracture using a texture analyzer (Perten TVT 6700, Stockholm, Sweden), fitted with a cylindrical probe 75 mm in diameter.

Eggshell ash, Ca, and P were determined on one egg collected on the same day from each focal bird at 50 WOA. The egg was broken open and the contents, including shell membranes, were removed. The eggshell was then gently washed, air dried and weighed with a digital scale before drying at 105°C for 24 h. It was then incinerated in a muffle furnace oven at 500°C for 8 h, then allowed to cool in a desiccator before the remaining ash was weighed. The percentage eggshell ash was calculated relative to eggshell air-dry weight. The eggshell composition of Ca and P were determined on the eggshell ash at The University of New South Wales as previously outlined for the analysis of diet Ca and P.

### Bone Quality

At 50 WOA 10 birds per treatment group were selected, weighed, and then euthanized by atlanto-cervical dislocation. Birds were selected for euthanasia to ensure they represented the range of bird performance within that treatment group while avoiding birds involved in egg quality assessment. For this all birds within one treatment group were stratified into high, medium, and low CFCR, then 3 birds were selected at random from the high and low CFCR range and 4 birds from the medium CFCR range for euthanasia.

Following bird euthanasia, the keel bone was exposed by retraction of the skin across the breast muscle. The curvature of the keel was scored in-situ on a four-point scale (Hy-Line International Technical Update, 2016). Keel curvature score 1 indicated a normal keel, score 2 a mild keel curvature, score 3 a moderate keel curvature and score 4 a severe keel curvature.

The left femur was then collected, frozen, and stored at −20°C until analysis. Before measurement, the femur was thawed to room temperature and the skin, ligaments and muscles were removed. Individual femur weight was measured using a digital scale and the length and external diameter at the mid-shaft was measured. Femur breaking strength (N) was then determined as the peak force to fracture at the mid shaft (horizontal plane) using a texture analyzer (Perten TVT 6700), fitted with a break probe (671170 break probe with a 675045 break rig set). All bones were held in the same orientation and the force was applied at the mid-length of the bone. The cortical thickness and medullary bone diameter were then measured at the breaking point using digital Vernier calipers with an accuracy of ± 0.01 mm. Bone density was measured as bone weight to bone length (Souza et al., 2017), modified to 100 g/mm index where higher bone density is indicated by higher weight to length index. The broken bones were later used to determine the femur ash content. For this the bones were dried at 105°C for 24 h then ignited to ash at 600°C for 8 h, cooled in a desiccator and weighed. The percentage femur ash was determined relative to the dry weight of the femur.

### Liver Health

Liver health was also assessed at 50 WOA on the same 10 birds/treatment group euthanased for keel curvature and femur assessment. The liver was observed in situ and evaluated for FLHS score as described by Shini et al. (2019) (scores ranged from 0 to 5; where 0 identified a liver of normal appearance without hemorrhage; 1 indicated a liver with 1–10 subcapsular petechial or ecchymotic hemorrhages; 2 identified a liver with more than 10 subcapsular petechial or ecchymotic hemorrhages while scores ≥3 indicate prominent hematomas and substantial liver hemorrhage together with a ruptured liver capsule). A sample of liver tissue was then snap-frozen in liquid nitrogen and stored at −80°C until assayed for lipid peroxidation through measurement of thiobarbituric acid reactive substances (**TBARS**). Here liver samples were thawed on ice, cut into small pieces and if necessary, washed twice with ice-cold phosphate buffered saline to remove any blood. Twenty-five mg of tissue was then transferred into a 2.0 mL safe lock tube containing two, 3-mm diameter metal beads. Two hundred and fifty µL radioimmunoprecipitation assay buffer with protease inhibitor (**EDTA**; 10 µL/mL) was added to each tube and the sample was lysed and homogenized using Qiagen TissueLyser II at a frequency of 30 for 2 min. The tube was then centrifuged at 16,000 × *g* for 10 min at 4°C to remove insoluble materials. The supernatant was collected and TBARS was measured using a Cayman TBARS assay kit (TCA Method, Item No. 700870) as described by the manufacturers (Cayman, Ann Arbor, MI).

### Statistical Analysis

Data were analyzed in a factorial design comprising 2 dietary treatments (HND and LND) × 2 18 WOA BW groups (HW and LW) using the generalized linear model procedure of STATISTICA (Statsoft Inc. 2003). The individual hen served as the experimental unit. Means were separated using the Tukey-Kramer method. All data are presented as means ± pooled SEM. The probability value which denotes statistical significance is *P* < 0.05.

## RESULTS

### Diet

Table 1 presents the experimental diet ingredients, formulated nutrient and energy levels and assayed gross energy, CP, crude fat, Ca and P. The ratio of the analyzed GE of HND and Early-lay LND diets (1.05) is lower than the ME ratio in the formulated diets (1.09). Crude protein of the mixed HND diet was 17.9% and the mixed Early-lay LND diet was 15.7% compared to formulated levels of 17.6 and 16.4%, respectively. The analyzed crude fat content was 3.1 and 2.1% for the HND and the Early-lay LND diet respectively, compared to formulated 4.92 and 2.54%. Analyzed Ca levels in the mixed diets were 5.4 and 6.2% in the HND and Early-lay LND diet respectively, and 0.57% and 0.40% total P respectively. In the diet formulation Ca was included at 3.981 and 4.212% in the HND and Early-lay LND diet respectively, and 0.556 and 0.445% total P. In the Mid-lay diet GE was 14.3 MJ/kg. Crude protein was 16.2% compared to formulated 16.0% and crude fat was 2.7% compared to formulated 2.5%. The analysed Ca and total P levels were 5.05 and 0.46% in the mixed diet and 4.29 and 0.42% in the diet formulation respectively.

### Body Weight and Production Performance

As required for the experimental design, at 18 WOA the mean BW of the HW birds (1.65 kg) was heavier (*P <* 0.001) than the LW birds (1.49 kg). Birds were identified as being HW or LW relative to ISA Brown breed product guide (ISA Brown Product Guide, Cage production system, 2017) recommended 18 WOA BW of 1.576 kg. There was no difference in 18 WOA mean BW for birds allocated to the HND diet (1.57 kg) compared to the LND diet (1.57 kg; *P* > 0.05; Table 2), matching the expected 18 WOA BSW. There was a 40 g difference between the BW of the lightest bird in the HW group and the heaviest bird in the LW group. Similarly, at 24 and 50 WOA average BW of HW (1.83 and 2.09 kg, respectively) and LW (1.70 and 1.88 kg, respectively) birds were different (*P* < 0.001). There was no effect of diet nutrient density on 50 WOA BW (Table 2), however, at 24 WOA, the end of the diet nutrient density treatment period, the HND diet fed birds had higher mean BW than LND diet fed birds (*P* < 0.001). There were no bird mortalities throughout the study.

**Table 2:** Hen weight and production.

Treatment	BW 18 woa (kg)	BW 24 woa (kg)	BW 50 woa (kg)	CFI^7^ 18-24 woa (kg)	CEP^8^ 18–24 woa	CEM^9^ 18–24 woa (kg)	CFCR^10^ 18–24 woa	CFI^7^ 18-50 woa (kg)	CEP^8^ 18–50 woa	CEM^9^ 18–50 woa (kg)	CFCR^10^ 18–50 woa
BW^1^ (18 woa)^2^											
HW^3^	1.65	1.83	2.09	5.16	44.6	2.33	2.27	26.8	222.6	13.0	2.07
LW^4^	1.49	1.70	1.88	4.86	42.7	2.15	2.35	24.8	218.9	12.4	2.00
SEM	0.01	0.01	0.02	0.03	0.48	0.28	0.06	0.17	1.05	0.1	0.02
Diet density											
HND^5^	1.57	1.79	1.98	4.98	43.8	2.31	2.19	25.7	221	12.8	2.01
LND^6^	1.57	1.74	1.99	5.04	43.5	2.17	2.43	26.0	221	12.6	2.06
SEM	0.01	0.01	0.02	0.03	0.48	0.28	0.06	0.17	1.05	0.1	0.02
Interaction											
HW × HND	1.65	1.85	2.09	5.10	44.6	2.37	2.18	26.6	223.0	13.1	2.04
HW × LND	1.66	1.81	2.09	5.22	44.7	2.29	2.35	27.0	222.1	13.0	2.09
LW × HND	1.50	1.73	1.87	4.86	43.0	2.25	2.20	24.7	218.9	12.6	1.97
LW × LND	1.49	1.67	1.89	4.86	42.4	2.05	2.51	24.9	218.9	12.3	2.04
SEM	0.01	0.01	0.03	0.05	0.68	0.40	0.08	0.25	1.48	0.14	0.02
*P*-value											
BW	0.0001	<0.0001	<0.001	<0.0001	0.005	<0.0001	0.290	<0.0001	0.014	<0.0001	0.005
Diet density	0.97	<0.001	0.66	0.19	0.69	<0.001	0.005	0.20	0.763	0.18	0.009
BW × diet density	0.13	0.64	0.78	0.25	0.60	0.12	0.404	0.55	0.779	0.42	0.76

1 BW, body weight.

2 woa, weeks of age.

3 HW, heavier body weight.

4 LW, lighter body weight.

5 HND, Early-lay higher nutrient density diet fed from 18 to 24 woa inclusive then Early-lay lower nutrient density diet fed from 25 to 39 woa followed by Mid-lay lower nutrient density diet fed from 40 to 50 woa.

6 LND, Early-lay lower nutrient density diet fed from 18 to 39 woa, then Mid-lay LND diet fed from 40 to 50 woa.

7 CFI, cumulative feed intake.

8 CEP, cumulative egg production.

9 CEM, cumulative egg mass.

10 CFCR, cumulative feed conversion ratio.

From 18 to 24 WOA there was an effect of BW on CFI such that the HW birds had consumed more than the LW birds (*P* < 0.001). Diet nutrient density however did not affect CFI during this same period (*P* = 0.19). Ultimately, the 18 to 50 WOA CFI remained higher (*P* < 0.001) in HW birds whose average total FI was 26.8 kg, compared to 24.8 kg for the LW birds (Table 2). There was no effect of diet nutrient density on 18 to 50 WOA CFI.

Across 18 to 24 and 18 to 50 WOA cumulative egg production (**CEP**) of HW birds was higher than with LW birds (*P* < 0.01 and *P* < 0.05 respectively), but diet nutrient density did not affect CEP. Similarly, CEM of HW birds was higher than LW birds between 18 and 24 WOA (2.33 kg vs. 2.15 kg; *P* < 0.001) and 18 to 50 WOA (13.0 kg vs. 12.4 kg; *P <* 0.001; Table 2). During 18 to 24 WOA diet nutrient density altered CEM, with birds on the HND diet achieving higher CEM (*P* < 0.001) compared to birds on the LND diet. This diet density treatment difference did not persist for 18 to 50 WOA. Cumulative FCR from 18 to 24 WOA was lower for the HND diet compared to the LND diet fed birds (2.19 and 2.43 respectively; *P* < 0.01) and remained lower for the HND diet birds through to 50 WOA (2.01 compared to 2.06, respectively; *P* < 0.01). Similarly, across 18 to 50 WOA CFCR was lower in LW compared to HW hens (*P <* 0.01) at 2.00 and 2.07 respectively.

### Egg Quality

The internal characteristics of eggs produced from focal birds from 46 to 50 WOA are presented in Table 3. Not surprisingly HW birds produced heavier eggs averaging 62.2 g (*P* < 0.05) compared to 60.1 g for LW birds. Haugh units were not impacted by BW nor diet nutrient density and were above 95. However, yolk diameter and yolk weight as percentage of EW were higher (*P <* 0.01; *P* < 0.05, respectively) in HW compared to LW hens, and concurrently the ratio of albumen to yolk was also lower with HW hens (*P* < 0.05). There was no effect of either BW nor diet nutrient density on albumen weight as a percent of EW. Yolk color score was not impacted by BW but, it was approaching significance due to diet nutrient density being higher in hens that had received the LND as opposed to the HND diet (*P* = 0.08; Table 3).

**Table 3:** Internal egg characteristics of egg weight, Haugh unit, yolk diameter, albumen weight, yolk weight, albumen: yolk and yolk color score from 46 to 50 wk of age.

Treatments	Egg weight (g)	HaughUnit	Yolk diameter (mm)	Albumen weight (%)	Yolk weight (%)	Albumen:yolk	Yolk color score^7^ (range 1–15)
BW^1^ (18 woa^2^)							
HW^3^	62.2	96.5	38.8	58.0	26.7	2.18	12.9
LW^4^	60.1	96.5	37.9	58.6	25.9	2.28	12.8
SEM	0.68	0.71	0.21	0.31	0.25	0.03	0.08
Diet density							
HND^5^	60.7	95.9	38.3	58.0	26.4	2.21	12.7
LND^6^	61.6	97.1	38.4	58.6	26.2	2.25	12.9
SEM	0.68	0.71	0.21	0.31	0.25	0.03	0.08
Interaction							
HW × HND	61.1	96.5	38.5	57.6	26.8	2.16	12.7
HW × LND	63.3	96.5	39.2	58.4	26.5	2.21	13.1
LW × HND	60.2	95.3	38.0	58.4	25.9	2.26	12.7
LW × LND	59.9	97.7	37.7	58.8	25.9	2.29	12.8
SEM	0.97	1.00	0.30	0.44	0.35	0.04	0.12
*P*-value							
BW	0.03	0.98	0.002	0.15	0.04	0.04	0.19
Diet density	0.35	0.22	0.56	0.19	0.63	0.36	0.08
BW × Diet density	0.19	0.23	0.09	0.67	0.61	0.69	0.19

1 BW, body weight.

2 woa, weeks of age.

3 HW, heavier body weight.

4 LW, lighter body weight.

5 HND, Early-lay higher nutrient density diet fed from 18 to 24 woa inclusive then Early-lay lower nutrient density diet fed from 25 to 39 woa followed by Mid-lay lower nutrient density diet from 40 to 50 woa.

6 LND, Early-lay lower nutrient density diet fed from 18 to 39 woa then Mid-lay LND diet fed from 40 to 50 woa.

7 Yolk color score: DSM color fan: 1 (palest) through to 15 (darkest) color scale.

The ESI was higher in birds that had received the LND diet from 18 to 24 WOA compared to the HND diet (*P <* 0.01). There was no effect of BW on ESI (Table 4). Shell weight as a percent of total EW and, shell thickness were both higher for LW birds, and both were approaching significance (10.8% compared to 10.5% *P* = 0.07; 0.4 mm compared to 0.39 mm *P =* 0.09 respectively), while diet nutrient density had no effect. No differences were observed in eggshell breaking strength and total shell ash, nor Ca content in the shell, however, shell P was higher in LW birds (1.3 g/kg vs. 1.1 g/kg; *P* < 0.01) compared to HW birds (Table 4).

**Table 4:** Egg shell characteristics at 46–50 wk of age.

Treatment	Egg shape index^7^	Shell weight^8^ (%)	Shell thickness (mm)	Shell breaking strength (N)	Total shell ash^9^ (%)	Ca^10^ (g/kg)	P^11^ (g/kg)
BW^1^ (18 woa^2^)							
HW^3^	77.1	10.5	0.39	43.4	94.2	383.4	1.1
LW^4^	76.7	10.8	0.40	45.4	94.0	368.6	1.3
SEM	0.56	0.09	0.005	1.14	0.17	8.6	0.04
Diet density							
HND^5^	75.7	10.7	0.40	45.5	94.2	370.5	1.3
LND^6^	78.1	10.7	0.39	43.3	94.1	381.5	1.2
SEM	0.56	0.09	0.005	1.14	0.17	8.6	0.04
Interaction							
HW × HND	75.8	10.6	0.39	43.5	94.3	376.0	1.2
HW × LND	78.5	10.6	0.39	43.3	94.2	390.7	1.1
LW × HND	75.7	10.8	0.41	47.5	94.1	364.9	1.3
LW × LND	77.7	10.8	0.39	43.3	94.0	372.3	1.3
SEM	0.79	0.13	0.007	1.6	0.24	12.1	0.06
*P*-value							
BW	0.61	0.07	0.09	0.22	0.47	0.23	0.02
Diet density	0.005	0.80	0.40	0.18	0.62	0.37	0.21
BW × diet density	0.65	0.81	0.12	0.21	0.86	0.76	0.34

1 BW, body weight.

2 woa, weeks of age.

3 HW, heavier body weight.

4 LW, lighter body weight.

5 HND, Early-lay higher nutrient density diet fed from 18 to 24 woa inclusive then Early-lay lower nutrient density diet from 25 to 39 woa followed by Mid-lay lower nutrient density diet fed from 40 to 50 woa

6 LND, Early-lay lower nutrient density diet fed from 18 to 39 woa then Mid-lay LND diet fed from 40 to 50 woa

7 Egg shape index, egg width divided by egg height multiplied by 100.

8 Shell weight: shell weight as a percent of egg weight.

9 Total shell ash, total shell ash as a percent of shell weight measured at 50 woa only.

10 Ca, calcium; measures taken at 50 woa only.

11 P, phosphorus; measures taken at 50 woa only.

### Bone Quality

At 50 WOA neither BW nor diet nutrient density had affected keel curvature, femur weight, femur length, femur weight to length index, medullary bone diameter, femur ash, and femur breaking strength (Table 5). However, there were interactions between BW and diet nutrient density for femur diameter (*P <* 0.05) and cortical thickness (*P* < 0.001). Femur diameter was highest for LW birds fed the HND diet (7.67 mm) and lowest for both LW birds fed the LND (7.26 mm) and HW birds fed the HND diet (7.27 mm) with HW birds on LND diet being intermediate (7.51 mm). The cortical bone thickness was also highest in the LW birds that had received the HND diet (0.9 mm) which was not different from the HW birds fed LND (0.87 mm) and, the BSW birds fed HND diet (0.85 mm). At 0.8 mm the LW birds on the LND diet had the narrowest cortical bone which was not different from HW birds fed the HND diet.

**Table 5:** Keel curvature scores and femur characteristics at 50 wk of age.

Treatment	Keel curvature (score 1-4)	Femur weight (g)	Femur length (mm)	Femur W:L index^7^	Femur diameter (mm)	Cortical thickness (mm)	Medullary Bone diameter (mm)		Femur bone ash (%)^8^	Femur breaking strength (N)
BW^1^ (18 woa^2^)										
HW^3^	2.37	11.4	84.6	13.5	7.39	0.86	6.29		47.1	203.7
LW^4^	2.25	11.0	84.0	13.1	7.46	0.85	6.33		47.5	195.8
SEM	0.21	0.20	0.46	0.24	0.11	0.01	0.09		0.79	10.5
Diet density										
HND^5^	2.37	11.1	84.3	13.2	7.47	0.87	6.35		46.9	201.7
LND^6^	2.25	11.3	84.3	13.4	7.38	0.84	6.26		47.7	197.7
SEM	0.21	0.20	0.46	0.24	0.11	0.01	0.09		0.79	10.5
Interaction										
HW × HND	2.54	11.2	85.0	13.2	7.27^b^	0.85^AB^	6.23		46.7	202.6
HW × LND	2.20	11.6	84.3	13.8	7.51^a,b^	0.87^A^	6.35		47.5	204.8
LW × HND	2.20	11.0	83.7	13.2	7.67^a^	0.90^A^	6.47		47.2	200.9
LW × LND	2.30	10.9	84.4	13.0	7.26^b^	0.80^B^	6.18		47.8	190.6
SEM	0.29	0.28	0.65	0.34	0.15	0.02	0.13		1.12	14.9
*P*-value										
BW	0.69	0.10	0.35	0.23	0.65	0.48	0.77		0.76	0.60
Diet density	0.69	0.56	0.97	0.85	0.59	0.03	0.51		0.52	0.79
BW × Diet density	0.46	0.40	0.30	1.00	0.04	0.001	0.12		0.94	0.68

^a,b,c^Columns without a common superscript are significantly different at *P* ≤ 0.05.

^A,B,C^Columns without a common superscript are significantly different at *P* ≤ 0.01.

1 BW, body weight.

2 woa, weeks of age.

3 HW, heavier body weight.

4 LW, lighter body weight.

5 HND, Early-lay higher nutrient density diet fed 18–24 woa inclusive then birds fed Early-lay lower nutrient density diet from 25 to 39 woa followed by Mid-lay lower nutrient density diet from 40 to 50 woa.

6 LND, Early-lay lower nutrient density diet fed from 18 to 39 woa then Mid-lay LND diet fed from 40 to 50 woa.

7 Femur W:L^+^; Modified femur weight:femur length index based on 100 g/mm.

8 Femur bone ash (%); bone ash weight as % of femur weight.

**Table 6:** Hen liver health at 50 weeks of age.

Treatment	FLHS^7^(range 0-5)	Liver lipid peroxidase (TBARS^8^, µM)
BW^1^ (18 woa^2^)		
HW^3^	1.70	0.64
LW^4^	1.05	0.56
Sem	0.21	0.01
Diet density		
HND^5^	1.00	0.55
LND^6^	1.75	0.65
sem	0.20	0.01
Interaction		
HW × HND	1.50	0.59
HW × LND	1.90	0.69
LW × HND	0.50	0.52
LW × LND	1.60	0.61
sem	0.29	0.02
BW	0.03	0.0003
Diet density	0.01	0.00002
BW × Diet density	0.24	0.920

1 BW, body weight.

2 woa, weeks of age.

3 HW, heavier body weight.

4 LW, lighter body weight.

5 HND, Early-lay higher nutrient density diet fed 18–24 woa inclusive then Early-lay lower nutrient density diet from 25 to 39 woa followed by Mid-lay lower nutrient density diet from 40 to 50 woa.

6 LND, Early-lay lower nutrient density diet fed from 18 to 39 woa then Mid-lay LND diet fed from 40 to 50 woa.

7 FLHS, fatty liver haemorrhagic syndrome score.

8 TBARS, thiobarbituric acid reactive substances.

### Liver Health

At 50 WOA FLHS scores were affected by both BW and diet nutrient density (Table 6). Heavier birds had higher FLHS scores compared to the LW birds (*P <* 0.05). Hens that had been fed the LND diet from 18 to 24 WOA had higher FLHS scores than those that had received the HND diet during that same time (*P <* 0.05). Similar differences were observed in the liver lipid peroxidase with HW birds having higher TBARS compared to LW birds (*P <* 0.001) and, the birds that had been fed the LND diet had higher TBARS than birds that had received the HND diet (*P <* 0.001).

## DISCUSSION

This study has demonstrated the responses of ISA Brown hens of different POL BW, that is HW and LW compared to BSW, fed diets of either LND or HND during early lay (18–24 WOA inclusive) on BW, CEM, CFI, and CFCR from 18 to 50 WOA, together with egg quality from 46 to 50 WOA and, their liver health and femur characteristics at 50 WOA. Differences between the 18 WOA BW of the HW (average 1.65 kg) and LW (1.49 kg) birds were inherent in the experimental design and purposely averaged above or below the ISA Brown breed standard recommended 18 WOA BW of 1.58 kg (ISA Brown Product Guide, Cage Production System, 2017). Hen weight was a central parameter in this study as it is known to impact layer hen ADFI, feed efficiency and egg characteristics (Harms et al., 1982; Leeson and Summers, 1987; Akter et al., 2019). Larger sized birds are frequently grown in Australia due to the commercial desire for larger sized eggs (Parkinson et al., 2008), while their physiological potential has been seen to be maximized around the BSW for age or, in slightly smaller hens (Parkinson et al., 2015). The smaller sized bird may also achieve good continuity of lay and cumulative egg production, egg size, and egg quality (Parkinson et al., 2015) and hence the comparison between HW and LW birds in this study.

It was not unexpected that the trajectory of hen BW followed on from the 18 WOA BW (Harms et al., 1982; Bish et al., 1985), such that average BW of HW birds was higher than LW birds at 24 and 50 WOA. The ISA Brown breed standard guide recommends that ISA Brown hens gain approximately 0.37 kg between 18 and 50 WOA (ISA Brown Product Guide, Cage Production System, 2017). At 0.39 kg and 0.44 kg weight gain respectively both the LW and particularly the HW birds gained more than breed standard recommendation during this time. As observed by Leeson and Summers (1987) in Leghorn pullets, the LW hens in this study did not demonstrate compensatory growth.

At the end of the diet nutrient density treatment period that is, end of 24 WOA, birds on the HND diet were heavier than those on LND diet. This is most likely due to the birds on the HND diet not adjusting their ADFI for the more nutrient dense diet and hence consuming a similar total amount of feed from 18 to 24 WOA as birds on the LND diet. This is at odds with Leeson et al. (2001); Perez-Bonilla et al. (2012); Ribeiro et al. (2014) and dePeriso et al. (2015) where Shaver White, Hy-Line Brown, White Dekalb, and Hy-Line W36 White Leghorn hens respectively adjusted ADFI with diet nutrient density. In these same experiments the Hy-Line Brown hens on the diet of the highest metabolizable energy level had higher BW gain compared to birds fed the diet of the lowest ME content (Perez-Bonilla et al., 2012) while Shaver White, White Dekalb and Hy-Line W36 White Leghorn hen weight was not affected by diet nutrient density. However, it must be noted that these observations in the Hy-Line W36 White Leghorn birds (dePersio et al., 2015) relate to the early laying period (19–26 WOA) only. As observed in our study, limited adjustment in ADFI in response to the diet nutrient density has been reported previously (Morris, 1968; Jalal et al., 2007). Our study is unique as the diet nutrient density treatment was applied in ISA Brown hens for a comparatively short period of time, from 18 WOA for the initial 7 wk during early lay only. The experimental design of studies involving diet nutrient density vary in several aspects including strain of bird, the initial bird age at which the diet nutrient density treatments were fed, the time frame over which the diets were fed and, the reported observations. For example, Shaver White received different diets from 19 WOA for twelve, 28-d sessions in Leeson et al. (2001); Hy-Line W-36 were fed different diets from 19 WOA to 70 WOA in dePersio et al. (2015); White Dekalb hens received dietary treatments from 23 WOA for 17 wk in Ribeiro et al. (2014); different diets were fed to Hy-Line Brown from 24 WOA to 59 WOA in Perez-Bonilla et al. (2012) and, four different layer strains were fed different diets from 22 WOA for 28 wk in Jalal et al. (2007). These distinctions in experimental design make it challenging to directly compare results. In the current study the early lay LND diet was formulated to 110 g ADFI and HND diet to 90 g ADFI. As ADFI of the HND diet was above 100 g/bird/day during 24 WOA these birds were moved onto the LND diet at 25 WOA. Body weight differences due to diet nutrient density which were apparent at 24 WOA, were no longer evident at 50 WOA.

A positive relationship between CFI and bird BW, as has been reported by others (Harms et al., 1982; Leeson and Summers, 1987; Perez-Bonilla et al., 2012) was also evident in this study, where HW bird 18 to 50 WOA CFI was 8% more than the LW bird CFI. At 50 WOA the ISA Brown HW birds were on average consuming an additional 4.5 g/d for each additional 100 g BW. An average change of 3.4 g feed for each 100 g change in BW has been reported for Leghorn pullets to 67 WOA (Leeson and Summers, 1987)*,* while Dekalb XL hens consumed on average an additional 6.7 g/d for each additional 100 g BW to 47 WOA (Harms et al., 1982). It should be noted that these comparisons involve different layer strains which are likely to have innate differences in FI as seen by Harms et al. (2000). As with the higher CEP of the HW compared to LW hens in this study, it could be expected that between 50-70% of the difference in FI of HW hens be attributed to their BW (bird maintenance), change in BW and EM (Fairfull and Chambers, 1984).

By the end of 24 WOA the HW birds had already achieved higher CEP compared to LW birds, and they continued with higher EP through to 50 WOA. Concurrently the HW birds also generated higher CEM from 18 to 50 WOA, a combination of their heavier EW and higher EP when compared to the LW birds. Similar higher EM, though not EP in heavier weight birds was observed by Perez-Bonilla et al. (2012). A simple cost benefit comparison of HW and LW bird CFI with CEP from 18 to 50 WOA in the current study is insightful as the HW hens produced an additional 3.7 eggs for an additional 2 kg of feed. While feed costs and return for eggs will vary at current Australian estimated cost of feed ($410 AUD/ton) and return per first grade egg ($0.13 AUD), to break even each HW bird needed to produce 6.3 more eggs for the additional 2 kg feed or, to consume only an additional 1.17 kg feed for the additional 3.7 eggs produced. While there were no differences in CEP due to diet nutrient density at 24 WOA, birds that had received the HND diet to 24 WOA had higher CEM to 24 WOA. This however did not continue to 50 WOA which is most likely the consequence of these birds moving to the LND diet at the start of 25 WOA.

In a similar vein and of particular interest is the improvement in CFCR to 24 and 50 WOA in birds that received the HND diet compared to birds that had continuously received the LND. Both Perez-Bonilla et al. (2012) and dePersio et al. (2015) also report improved efficiency of converting feed to eggs in birds receiving a higher energy diet. In terms of BW treatment, no differences in CFCR were observed across 18 to 24 WOA but LW birds achieved lower CFCR for the longer 18 to 50 WOA period. Interestingly no impact of BW on FCR (kg feed/kg eggs) was found with Hy-Line Brown hens during 24 to 59 WOA (Perez-Bonilla et al., 2012) but Harms et al. (1982) report improved FE in LW Dekalb XL pullets across 31 to 47 WOA as did Akter et al. (2019) with ISA Brown hens between 35 and 41 WOA.

Hen weight is known to impact egg characteristic (Harms et al., 1982; Leeson and Summers, 1987; Leeson et al., 1997; Perez-Bonilla et al., 2012), which was reiterated in this study. The HW birds produced heavier eggs which had a wider yolk diameter, heavier yolk and lower albumen to yolk ratio, compared to LW birds. Greater yolk weight could be attributed to the higher energy intake of HW birds and, their opportunity to deposit a larger proportion of yolk into the egg. However, higher albumen to yolk ratio in eggs of LW hens at 50 WOA may be appealing to the consumer as a smaller yolk can represent lower cholesterol (Jiang and Sim, 1991). Diet nutrient density did not significantly impact egg characteristics at 50 WOA, though birds on LND diet treatment had tendency for darker yolk color than birds that had received the HND diet. Perez-Bonilla et al. (2012) observed the opposite with higher pigmentation in the yolk of eggs from hens that had been on a higher energy diet. They proposed that as xanthophyll pigment additives are fat soluble the pigments may be more readily absorbed in the higher energy diet. The HND diet of this study was formulated with close to double the fat content of the LND diet, but yolk pigmentation did not follow the same trend. These contrary observations may be attributed to the yolk color assessment in this study occurring 20 wk after the conclusion of feeding the HND. Additionally, the higher yolk color in birds fed the LND diet may be due to innate features of the individual hen or, due to the slightly higher overall FI to 50 WOA of LND diet and consequently a higher intake of color pigment (Karunajeewa et al., 1984). Average Haugh units were similarly high (above 90) in all treatments irrespective of BW or diet nutrient density. In contrast Perez-Bonilla et al. (2012) found significantly lower Haugh units in higher energy diets at a comparable 44 to 47 WOA, with the average Haugh unit for all diets being below 90. As with yolk pigmentation, the cessation of feeding HND diet at end of 24 WOA in this study as opposed to its ongoing provision in Perez-Bonilla et al. (2012) may contribute to these differences.

The 78.1 ESI from the LND diet indicates more rounded eggs compared to eggs from birds fed HND diet (ESI 75.7), the latter falling within the ideal ESI range of 72 to 76 (Duman et al. 2016). Not surprisingly the smaller eggs from the LW birds had a numerically higher proportion of shell which was also thicker than the shell of heavier eggs from HW birds. The percent shell was 10.5 or greater in all treatments with no differences in eggshell breaking strength. This aligns with findings of Abdallah et al. (1993) where less than 9.5% shell weight resulted in a rapid increase in cracked shells while at 10% or more shell weight, the frequency of shell fractures declined.

Interestingly at 50 WOA the eggshell of LW birds had higher P but similar Ca levels compared to the HW birds. Phosphorous is deposited in the vesicles of the eggshell cuticle and, in the outer shell, with the rate of deposition increasing towards the end of eggshell formation (Cusack et al., 2003). Cusack et al. (2003) also identified higher P in the outer eggshell of older (56 WOA) compared to younger (28 and 42 WOA) broiler breeders which may contribute to the maintenance of their eggshell quality. In the current study a numerically higher percent shell weight, shell thickness and shell breaking strength occurred concurrently with higher shell P in eggs from LW birds. Whether there is an association between eggshell P and eggshell quality in 50 WOA LW ISA Brown layer hens requires more extensive investigation.

The interaction of BW and diet nutrient density on femur diameter and cortical thickness at 50 WOA followed similar trends. In both cases the LW HND treatment resulted in higher, while the LW LND diet birds generated the lowest femur diameter and thinnest cortical bone. The thicker cortical bone of the LW HND diet treated birds compared to LW LND diet suggests that the HND diet may contribute to a thicker cortical bone in LW birds. In comparison the HW birds had similar cortical bone thickness irrespective of diet. The HND diet contained less Ca but higher P than the LND diet (Table 1) and it appears that the HND may play a role in reducing the exposure of structural bone in meeting Ca requirements for eggshell (Korver, 2020) through to 50 WOA in LW hens. Taylor and Moore (1958) proposed that the P involved in the rapid development of the ovary and oviduct and, calcification of the medullary bone at sexual maturity is drawn from the cortical bone. While limited bird numbers were involved, they also observed that a diet higher in P resulted in higher P in the cortical bone. In the current study the demand for P during sexual maturity may have been offset by the higher available P of HND diet for LW birds that were not in lay at 18 WOA (64% of birds – data not shown) when first fed the HND diet. This hypothesis clearly requires in-vivo evaluation. However, there were no differences in medullary bone diameter and, despite the differences in cortical bone thickness, no differences in femur ash or breaking strength. Similar femur breaking strength and keel curvature across treatments may reflect comparably low mobilization of structural bone-derived Ca for eggshell formation (Whitehead and Fleming, 2000). While it is tempting to link the similar femur breaking strength with eggshell strength between the treatment groups, no direct relationship has been established between EP and shell quality with bone strength (Jansen et al., 2020; Alfonso-Carrillo et al., 2021)

The FLHS and liver lipid peroxidase levels were both higher for the HW compared to LW birds and, birds that had received the LND compared to HND diet throughout the early laying phase. Being a disorder of liver lipid metabolism (Yang et al., 2017) FLHS is a multifactorial condition of caged layers under high demand for lipid processing. Layer hens of higher feed (Shini et al., 2020a) and energy (Yang et al., 2017) intake and heavier BW (Shini et al., 2019) are more susceptible to developing FLHS. This was also observed with the HW birds in this study. It is interesting that birds that had received the LND diet compared to HND diet from 18 to 24 WOA had higher FLHS scores but, based on formulated dietary ME and total FI of the different diets, all birds had a similar total energy intake across 18 to 50 WOA period. This suggests that other factors may have contributed to the higher FLHS scores in LND diet treated birds. Plasma estrogen, which is associated with FLHS (Shini et al., 2020b) was also not different between the birds at 50 WOA (data not shown). However, the higher levels of lipid peroxidase (TBARS) identified in the birds of higher FLHS scores corresponds with other findings (Squires and Wu, 1992; Zhang et al., 2008) and may be involved in FLHS.

## CONCLUSIONS

It can be concluded that the heavier weight ISA Brown hens had higher cumulative feed intake, cumulative egg production and cumulative egg mass but poorer cumulative feed conversion ratio from 18 to 50 WOA compared to lighter weight hens. Heavier weight hens also produced heavier eggs with higher yolk content but lower shell weight and thinner shells. They also experienced higher fatty liver hemorrhagic syndrome scores and lipid peroxidation compared to lighter weight birds. Modifying the flock's diet and feeding a more nutrient dense diet during early lay significantly benefited cumulative feed conversion ratio and increased cortical bone thickness, especially in lighter weight hens but, did not impact femur breaking strength. Feeding a higher nutrient dense diet during early lay also reduced hepatic lipid peroxidation and scores of fatty liver hemorrhagic syndrome. Therefore, offering lighter weight pullets a more nutrient dense diet during early lay provides an opportunity to prepare them for improved overall feed conversion efficiency and hen liver health through to 50 wk of age. The potential for similar benefits throughout a longer laying cycle from dietary treatments in early lay requires investigation.
